# Lower Risk of Stroke after Deformity Surgery: Long Term Benefit Demonstrated by a National Cohort Study

**DOI:** 10.3390/ijerph121012618

**Published:** 2015-10-12

**Authors:** Liang-Chung Huang, Wu-Fu Chung, Shih-Wei Liu, Peng-Yuan Chang, Li-Fu Chen, Jau-Ching Wu, Yu-Chun Chen, Wen-Cheng Huang, Laura Liu, Henrich Cheng, Su-Shun Lo

**Affiliations:** 1 Department of Emergency Medicine, National Yang-Ming University Hospital, I-Lan 260, Taiwan; E-Mails: horus7855@yahoo.com.tw (L.-C.H.); wolfchung2001@yahoo.com.tw (W.-F.C.); shihweiliu123@gmail.com (S.-W.L.); 2 School of Medicine, National Yang-Ming University, Taipei 112, Taiwan; E-Mails: acidbummer@gmail.com (P.-Y.C.); wchuang@vghtpe.gov.tw (W.-C.H.); hc_cheng@vghtpe.gov.tw (H.C.); sslo@ymuh.ym.edu.tw (S.-S.L.); 3 Department of Neurosurgery, Neurological Institute, Taipei Veterans General Hospital, No. 201, Shih-Pai Road, Sec. 2, Beitou, Taipei 11217, Taiwan; 4 Department of Medical Research and Education, National Yang-Ming University Hospital, I-Lan 260, Taiwan; 5 Institute of Hospital and Health Care Administration, School of Medicine, National Yang-Ming University, Taipei 260, Taiwan; 6 Department of Ophthalmology, Chang Gung Memorial Hospital, Taoyuan 333, Taiwan; E-Mail: lauraliu@cgmh.org.tw; 7 College of Medicine, Chang Gung University, Taoyuan 333, Taiwan; 8 Institute of Pharmacology, National Yang-Ming University, Taipei 112, Taiwan

**Keywords:** adult spinal deformity (ASD), spinal fusion surgery, stroke, National Health Insurance Research Database (NHIRD)

## Abstract

*Objectives*: This study aimed to investigate the long-term risk of stroke in adult patients with spinal deformity. Specifically, the study addressed the possible protective effect of surgery for spinal deformity against stroke. *Methods*: Using the National Health Insurance Research Database (NHIRD), a monopolistic national database in Taiwan, this retrospective cohort study analyzed the incidence of stroke in patients with adult spinal deformity (ASD) in a 11-year period. A total of 13,503 patients, between 55 and 75 years old, were identified for the diagnosis of ASD. The patients were grouped into two: the surgical group (*n* = 10,439) who received spinal fusion surgery, and the control group (*n* = 2124) who received other medical treatment. The incidence rates of all subsequent cerebrovascular accidents, including ischemic and hemorrhagic strokes, were calculated. Hazard ratios for stroke were calculated use a full cohort and a propensity score matched cohort. Adjustments for co-morbidities that may predispose to stroke, including hypertension, diabetes mellitus, arrhythmia and coronary heart disease were conducted. Kaplan-Meier and Cox regression analyses were performed to compare the risk of stroke between the two groups. *Results*: During the total observation period of 50,450 person-years, the incidence rate of stroke in the surgical group (15.55 per 1000 person-years) was significantly lower than that of the control group (20.89 per 1000 person-years, *p* < 0.001). Stroke was more likely to occur in the control group than in the surgical group (crude hazard ratio 1.34, *p* < 0.001; adjusted HR 1.28, *p* < 0.001, by a propensity score matched model). *Conclusions*: In this national cohort of more than 13,000 ASD patients covering 10 years, stroke was approximately 25% less likely to happen in patients who underwent spinal fusion surgery than those who received medical management. Therefore, spinal fusion surgery may provide a protective effect against stroke in adult patients with spinal deformity.

## 1. Introduction

Surgical management of adult spinal deformity (ASD) has become widely accepted in modern spine care. Various surgical approaches have demonstrated their effectiveness in the relief of clinical symptoms and the improvement of patients’ quality of life [1,2,3,4,5]. However, there is a paucity of data on the long-term influences of these patients’ medical conditions. Since spinal deformity can be very debilitating, it is intuitive to infer that restoration of lordosis, correction of scoliosis and kyphosis are correlated with improved general health. Nonetheless, whether the spinal surgery attenuates the long-term risk of medical comorbidities in these patients remains uncertain.

Cerebrovascular accident or stroke is the second leading cause of death worldwide [6]. Patients with degenerative scoliosis are commonly older and have substantial risk of many chronic medical diseases, such as cardiovascular, metabolic, cerebrovascular and rheumatic conditions [7,8]. Previous studies have demonstrated that patients who received spinal fusion and vertebroplasty have a similar risk of stroke [9,10,11]. It is not clear how these spinal operations affect the risk of stroke in elderly patients with scoliosis, especially since the surgery for spinal deformity is more complex and costly. Whether surgery for spinal deformity can lower the risk of medical catastrophes or improve the patients’ general health is an issue of public health.

This study aimed to investigate the long-term risk of stroke in adult patients with spinal deformity. Specifically, the study addressed the possible protective effect of surgery for ASD against stroke. In order to maximize case numbers and the duration of follow up, the authors used the National Health Insurance Research Database (NHIRD) of Taiwan, which is a monopolistic database operated by the government. This comprehensive database, containing more than 22 million insurants, covers over 99% of the population and financed healthcare for the entire population of Taiwan. The universal insurance system provides unrestricted access to health care, so that the NHIRD allows comprehensive follow up of deformity patients and precise calculation of the incidence of stroke.

## 2. Materials and Methods

### 2.1. Database, Registrations, and Standard Protocol Approvals

The NHIRD of Taiwan is provided by the National Health Research Institute (NHRI), which is composed of universal medical claims made by the Taiwanese population during the study period. The data have already undergone a de-identification and encryption process for the purpose of medical research. Individual patients or health service providers cannot be traced in the database because individual and hospital identifiers are unique to the research database and researchers. Therefore, this study was exempt from full review by the Institutional Review Board of the Taipei Veterans General Hospital (IRB# 2012-10-008BC). Moreover, the Bureau of National Health Insurance of Taiwan regularly performs cross-check and validation processes of the medical charts and claims, which ensure the accuracy of diagnosis coding in the NHIRD.

### 2.2. Hospitalization for ASD and Spinal Fusion Surgery

The International Classification of Disease, 9th Version (ICD-9) is used in the NHIRD to record all diagnoses of every admission. During the study period, for patients aged between 55 to 75 years, all hospitalizations discharged with the diagnostic code of 737.x and 738.5 were identified as ASD patients.

Patients with ASD could only be enrolled in this study if they had no previous stroke events recorded in the NHIRD from 1997 to 2000. The incidences of hospitalization for ASD were identified as subjects who were followed-up for more than one year and newly-hospitalized with the above mentioned discharge code of ASD between 1 January 2000 and 31 December 2010. The incidence rates in the study were estimated by the incidence density. Spinal fusion surgery was determined by occurrence of the procedure codes of spinal fusion (ICD-9 procedure codes, 81.04–81.08) during admission. These procedure codes included thoracolumbar spinal arthrodesis from anterior or posterior. All common surgical procedures for ASD were theoretically included (e.g., anterior, posterior, and combined anterior and posterior approaches). In order to include patients with primary ASD, patients had been diagnosed with the following conditions such as malignancies (ICD-9:140–239), sickle cell disease (282.6), thrombophilia (286.9), congenital heart defects (745–747), connective tissue defects (SLE: 710, RA: 714, scleroderma: 701.0–710.1, Sjögren’s syndrome: 710.2), Marfan syndrome (759.82) and Ehlers-Danlos syndrome (756.83) were excluded.

### 2.3. Surgical vs. Control Group

All of the identified ASD subjects were divided into two groups. The surgical group was composed of subjects who underwent any one of the above mentioned surgical procedures after the index date. The control group was composed of subjects who did not receive the above mentioned surgical procedures. Prior admission for stroke was determined by hospitalization records with corresponding discharge diagnostic codes (ICD-9 codes, 430-5.x) before the index date. After exclusion of all previous stroke and multiple/repeated operations, all subjects with ASD were followed up for subsequent events of stroke.

### 2.4. Covariates and Outcomes

Co-morbidities, which included hypertension (ICD-9 code, 401-5.x), diabetes mellitus (250.x), arrhythmia (426-7.x), coronary heart disease (410-4.x), and dyslipidemia (272.0-4) were designated as covariates. These were determined by the presence of either diagnostic codes in outpatient records or discharge codes of hospitalization records six months before the index date to the date of outcome event or the end of follow-up.

The study endpoint was the event of stroke-related hospitalization determined by the hospitalization records with discharge diagnostic code of stroke (ICD-9 code, 430-5.x). Furthermore, hemorrhagic strokes (430-2.x) *vs.* ischemic strokes (433-5.x) were also stratified for subgroup analysis. Every subject with ASD was followed-up for up to one year after the index date, or unless he/she had a stroke during the observation period.

### 2.5. Statistical Analysis

All of the data were linked using the SQL server 2008 (Microsoft Corp., Albuquerque, NM, USA) and analyzed using SPSS software (SPSS, Inc., Chicago, IL, USA). Chi-square and independent *t*-tests were used to assess differences in age, gender and co-morbidities between the surgical and control groups. Subsequently, the Kaplan-Meier method and Log-rank test were used to estimate and compare the incidence rates of hospitalization for stroke. Both the full cohort model and the propensity score matched model were used to compare the incidence rates of stroke between the two groups after adjustment for the aforementioned covariates (e.g., age, gender, Charlson’s comorbidity score, hypertension, diabetes, arrythmia, coronary heart disease, and dyslipidemia). A two-tailed level of 0.05 was considered statistically significant.

## 3. Results

**Figure 1:**
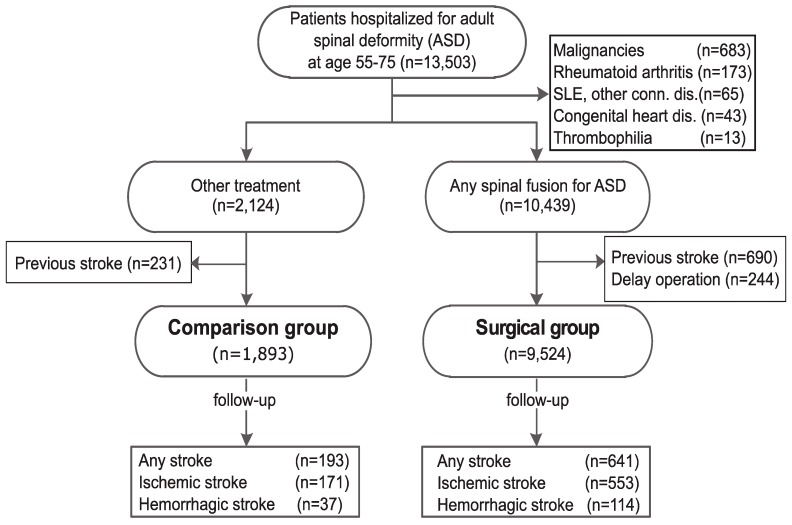
Data flow for patients hospitalized for adult spinal deformity (ASD) during 2000 and 2009.

Between 2000 and 2009, a total of 13,503 patients were hospitalized for ASD, with a total follow-up of 50,450 person-years. After exclusion of patients with previous stroke, there were 9524 patients (full, unmatched cohort) in the surgical group, and 1893 patients in the control group (Figure 1). The full cohort was further matched by a propensity score at a ratio of 1:1, named as the propensity score matched cohort, as demonstrated in Table 1.

### Incidence of Stroke in ASD Patients

The overall incidence of stroke-related admission in these ASD patients was 16.53 per 1000 person-years. Patients in the control group, those with ASD but who did not receive surgery, were more likely to be hospitalized for stroke than those in the surgical group. The incidence rate of stroke in the control group (20.89 per 1000 person-years) was 1.34 times higher than that in the surgical group (15.55 per 1000 person-years, *p* < 0.001). The crude hazard ratio for stroke was 1.34 (95% C.I., 1.14–1.58, *p* < 0.001). After adjusting for co-morbidities, the adjusted hazard ratio was 1.19 (95% C.I., 1.01–1.39, *p <* 0.05, estimated using a full cohort model) and 1.28 (95% C.I., 1.02–1.60, *p <* 0.05, estimated using a propensity score matched model), which suggested a significantly higher incidence rate of stroke in the control group than in the surgical group (Table 2).

**Table 1:** Characteristics of study cohort and propensity score matched cohort.

	Full Cohort (Original Unmatched Cohort)					Propensity Score Matched Cohort (Ratio 1:1)				
	Comparison Group		Surgical Group		*p*-Value	Comparison Group		Surgical Group		*p*-Value
	*n* = 1983	(%)	*n* = 9524	(%)		*n* = 1872	(%)	*n* = 1872	(%)	
Demographic characteristics										
Gender					<0.001					0.475
Female	1193	(60.2)	6480	(68.0)		1184	(63.2)	1205	(64.4)	
Male	700	(35.3)	3044	(32.0)		688	(36.8)	667	(35.6)	
Age, Mean (SD)	67	(6.0)	66.4	(5.7)	<0.001	67.0	(6.0)	67.3	(5.5)	0.083
Charlson’s score (SD)	1.1	(1.5)	0.7	(1.3)	<0.001	1.0	(1.4)	1.0	(1.5)	0.732
Comorbidities										
Hypertension					0.879					0.534
Yes	635	(32.0)	3212	(33.7)		622	(33.2)	640	(34.2)	
No	1258	(63.4)	6312	(66.3)		1250	(66.8)	1232	(65.8)	
Diabetes					0.402					0.965
Yes	318	(16.0)	1526	(16.0)		308	(16.5)	309	(16.5)	
No	1575	(79.4)	7998	(84.0)		1564	(83.5)	1563	(83.5)	
Arrythmia					<0.001					0.698
Yes	144	(7.3)	496	(5.2)		131	(7.0)	125	(6.7)	
No	1749	(88.2)	9028	(94.8)		1741	(93.0)	1747	(93.3)	
Coronary heart disease					<0.001					0.795
Yes	333	(16.8)	1065	(11.2)		318	(17.0)	324	(17.3)	
No	1560	(78.7)	8459	(88.8)		1554	(83.0)	1548	(82.7)	
Dyslipidemia					<0.001					0.812
Yes	163	(8.2)	590	(6.2)		153	(8.2)	157	(8.4)	
No	1730	(87.2)	8934	(93.8)		1719	(91.8)	1715	(91.6)	
Outcome										
Any stroke	193	(9.7)	641	(6.7)	<0.001	191	(10.2)	132	(7.1)	<0.001
Ischemic stroke	171	(8.6)	553	(5.8)	<0.001	169	(9.0)	117	(6.3)	0.001
Hemorrhagic stroke	37	(1.9)	114	(1.2)	0.008	37	(2.0)	24	(1.3)	0.093

**Table 2:** Incidence rate and hazard ratios for hospitalized for stroke.

	Total Sample (Per 1000 Person-Year)	Comparison Group (Per 1000 Person-Year)	Surgical Group (Per 1000 Person-Year)	
Incidence of hospitalized for stroke	16.53	20.89	15.55	
Number of hospitalized for stroke	834	193	641	
Observed person-years	50,450.10	9240.80	41,209.30	
Crude hazard ratio (95% C.I.)		1.34	1.00	(1.14–1.58) ***
Adjusted hazard ratio, full cohort (95% C.I.) **^a^**		1.19	1.00	(1.01–1.39) *
Adjusted hazard ratio, propensity score matched cohort (95% C.I.) **^b^**		1.28	1.00	(1.02–1.60) *

**^a^** hazard ratio was estimated by a full cohort model (*n* = 11,507) adjusted for age, gender, Charlson’s comorbidity score, hypertension, arrythmia, dyslipidemia; **^b^** hazard ratio was estimated by a propensity score matched model where comparison group and surgical group were matched by propensity score (calculated using age, gender, Charlson’s comorbidity score, hypertension, arrythmia, dyslipidemia); Significance level: ***: *p* < 0.001, *: *p* < 0.05.

**Figure 2:**
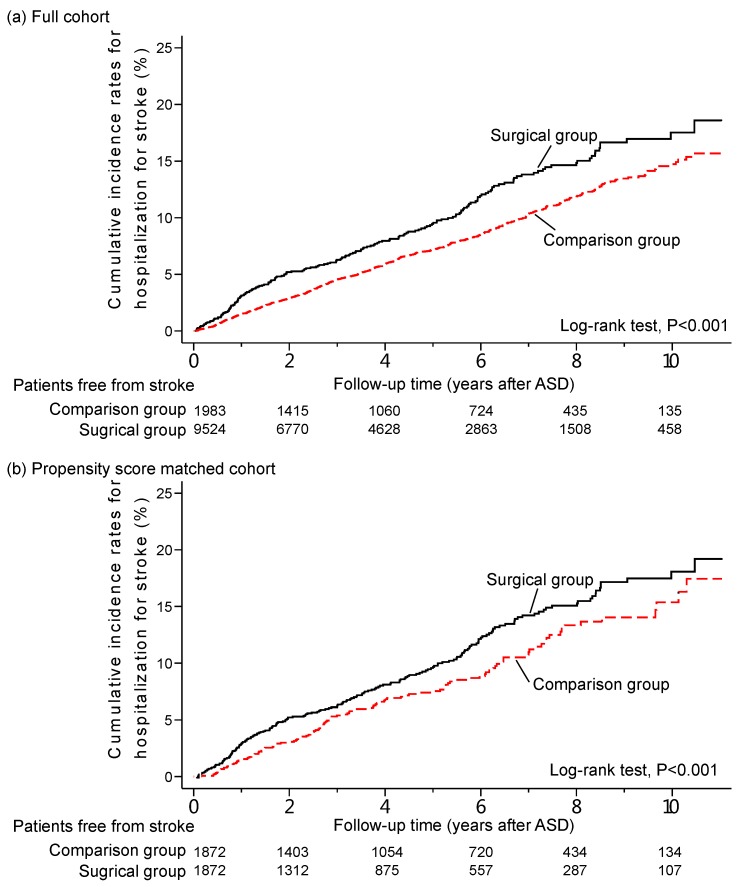
Cumulative incidence rates for stroke for patients hospitalized for adult spinal deformity (ASD) between 2000 and 2009. (**a**) Full cohort; (**b**) Propensity score matched cohort.

The Kaplan-Meier analysis demonstrated that the surgical group had significantly lower risk of stroke than the control group (Figure 2).

## 4. Discussion

To date, this study, comprising 13,503 subjects, was the largest, and the first on a national scale, of ASD patients, analyzing the incidence of subsequent stroke after surgical or non-surgical management, respectively. This comprehensive nationwide cohort with a total observation period of a decade demonstrated a lower risk of stroke in patients with ASD who received spinal fusion surgery. For those non-surgically managed patients, stroke was nearly two times more likely to happen. Therefore, ASD patients treated without surgery should be cautioned for subsequent stroke and strategies to prevent stroke could be considered.

The reason for the lower risk of stroke after deformity surgery remains uncertain. One possible explanation of this phenomenon is the increase in physical activity after a successful operation. In the literature, many reports have correlated increased physical activity with a lower risk for stroke [12,13,14]. For example, using a meta-analysis Lee *et al.* concluded that moderate or high levels of physical activity were associated with reduced risk of stroke [15]. Hu *et al.* demonstrated that moderate-intensity physical activities reduced risk of total and ischemic stroke in a dose-response manner [16]. Sattelmair *et al.* also reported that walking decreased the risk of total, ischemic and hemorrhagic strokes [17]. Even milder physical activities (e.g., daily commuting and leisure time activities) were reported to reduce the risk of stroke [18,19]. According to the guidelines of the American Heart Association (AHA) and American Stroke Association (ASA), physical activity is a well-documented modifiable risk factor for a first stroke and regarded as a primary prevention for ischemic stroke [20,21]. Patients with ASD may limit their physical activity due to a limitation in their range of spinal motion and the pain caused by undertaking more physical activity. These causes can be eliminated by surgical correction of the deformity and the rehabilitation programs in which patients enroll after surgery. It is reasonable to speculate that the decrease in the incidence of stroke after correction of spinal deformity in patients with ASD is due to the increased physical activity after surgery. Since there is an obvious hindrance to physical activity associated with debilitating ASD, the increased risk of stroke in those non-operated ASD patients can therefore be anticipated. However, this study does not directly support the association of stroke and physical inactivity.

The survival analysis in the present study clearly demonstrated the differences of stroke events between the surgical group and the control group. The divergence gradually increases in a steady fashion (Figure 2). The beneficiary effect of surgery for patients with ASD can therefore be expected in a greater magnitude when the observation time is longer. However, there is some inherent difficulty in such a cohort study to demonstrate greater differences in the Kaplan-Meier analysis. Due to the relatively old age of this cohort, between 55 and 75 years, there is this issue of competing risks of mortality. This can only be adjusted for by the inclusion of more medical co-morbidities and precise verification of cause of mortality. The major known risk factors of stroke, including hypertension, cardiac arrhythmia, diabetes, coronary heart disease and dyslipidemia, are already included and adjusted. Therefore, the estimation of the incidence of stroke as well as the association appeared sound and accurate.

There are several limitations to the present study. First, selection bias could exist within and between the control and the surgical groups. Patients who were managed with surgery could be different (had fewer risk factors of stroke) from those who were managed medically. It is possible that some patients were too ill to undergo deformity surgery and thus inherently were at higher risk of stroke. Second, the immediate risk of surgical complications, such as infection and flailed back syndrome, was not considered in the present study. The various surgical approaches, degree of correction, fusion length and rates were not taken into consideration. Furthermore, the heterogeneous nature of ASD, including the type and severity of scoliosis, compliance of patients with comorbidities to their treatment and the patients’ deformities or clinical outcome scores, should affect the clinical outcome of surgery and thus change the effect on long term results. However, this simplified data, with a large number of patients and a comprehensive follow up of all subsequent events of stroke, could overcome the problems and provide valuable information. The incidences of hospitalization for ASD, surgery for ASD, and stroke were precisely calculated through this national database. The correlation and risk analysis were therefore very close to the reality.

To date, there has been no report addressing the long-term medical benefit of surgery for ASD. Although there are many papers demonstrating the radiological and clinical effectiveness of surgery, little is known about its effect on the patients’ general health and chronic medical condition. The current study, for the first time, successfully demonstrated the protective effect of surgery for ASD patients. Despite the complexity of the surgery *per se* and the associated complications, patients who did not receive surgery for ASD had higher risk of stroke than those who had surgery.

## 5. Conclusions

In this national cohort of more than 13,000 ASD patients covering 11 years, stroke was approximately 25% less likely to happen in patients who underwent spinal fusion surgery than those who received medical management. Therefore, spinal fusion surgery may provide a protective effect against stroke in adult patients with spinal deformity.
